# Late Occurring Medial Migration of a Lag Screw in Gamma Nailing

**DOI:** 10.1155/2016/5201674

**Published:** 2016-05-23

**Authors:** S. van Hoef, M. C. H. W. Fuchs, R. H. M. ten Broeke

**Affiliations:** Department of Orthopaedic Surgery, Maastricht University Medical Center, P. Debyelaan 25, 6229 HX, Maastricht, Netherlands

## Abstract

An 81-year-old female was treated for a pertrochanteric multifragmentary fracture of the proximal femur with a third-generation Gamma nail. After 3 months she presented herself again with acute pain and inability to bear weight on the leg. Radiographs showed medial migration of the lag screw. She was treated with a total hip arthroplasty, after which she was successfully discharged. In this case report the possible causes of this late and unusual complication are discussed.

## 1. Introduction

As the average age of the general population increases so does the incidence of hip fractures [1]. Approximately half of the hip fractures are extra capsular [2], in which fixation with a dynamic hip screw or intramedullary fixation becomes the most favorable treatment [3]. Treatment with a Gamma nail is therefore a frequently used method in trauma surgery.

Despite being the most favorable treatment, complications do occur [4]. In our case we describe the rare complication of medial migration of the lag screw, three months after implantation of the Gamma nail.

## 2. Case Presentation

An 81-year-old woman was presented in the emergency department (ED) after falling on her left hip. She complained from severe pain in the left hip and inability to bear weight on her left leg. Clinical examination showed a shortened left leg in external rotation; all movements as well as axial pressure were painful.

**Figure 1:**
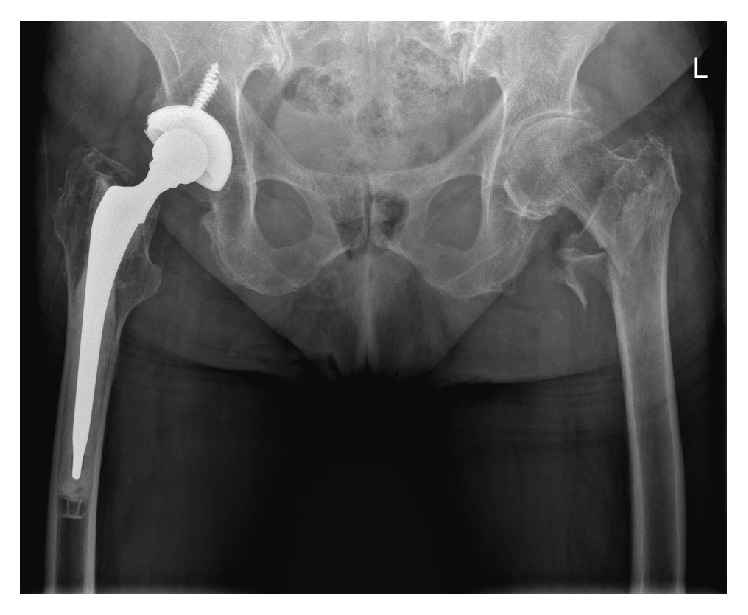
Anteroposterior X-ray showing a pertrochanteric fracture of the left femur.

Radiographs of the left hip, anterior-posterior and axial, showed a pertrochanteric multifragmentary fracture of the proximal femur (AO Classification 31-A2) (Figure 1).

**Figure 2:**
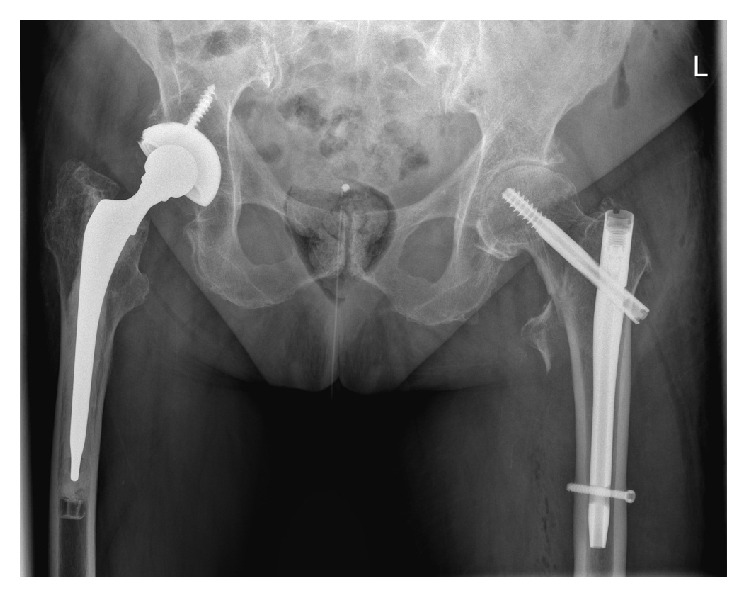
Anterior-posterior postoperative X-ray showing good positioning of the intramedullary Gamma nail in the right femur, with a tip-apex distance below 25 mm combined (AP+ axial).

**Figure 3:**
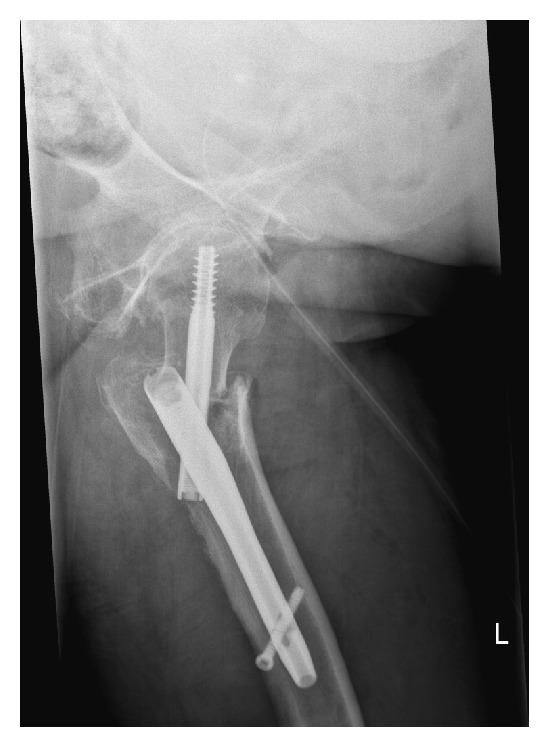
Axial postoperative X-ray showing good positioning of the intramedullary Gamma nail in the right femur.

The next day the patient was operated on where the fracture was fixed with a third-generation short Gamma nail (Stryker, Kalamzoo, USA), using a set screw to control rotation of the lag screw. The procedure and postoperative course were uneventful, with the Gamma nail in good position as could be confirmed on the postoperative X-ray (Figures 2 and 3). The patient was able to mobilize and was allowed full weight bearing of the left leg the next day. After 11 days the patient was discharged to a rehabilitation center.

**Figure 4:**
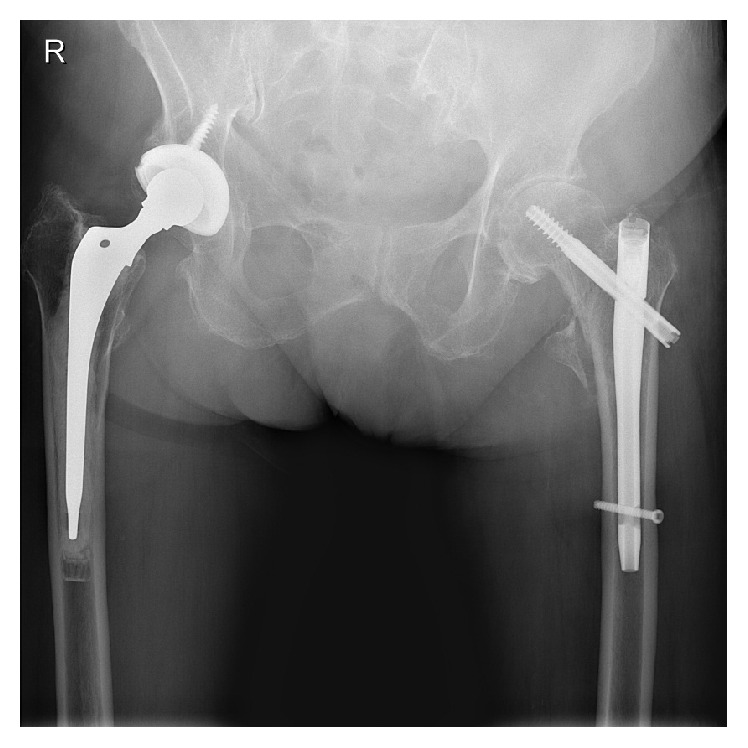
Anterior-posterior X-ray 6 weeks after operation, showing unchanged position and signs of consolidation.

After six weeks we saw the patient in the outpatient clinic for a scheduled follow-up. At that point there were no complaints and she was mobilizing with a walker. The X-ray after 6 weeks (Figure 4) showed an identical position of the Gamma nail and consolidation of the fracture.

**Figure 5:**
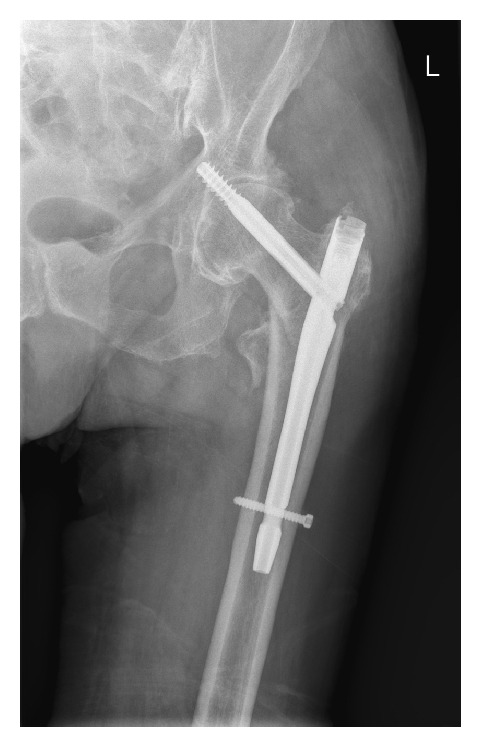
Anterior-posterior X-ray showing medial migration of the lag screw into the acetabulum after 12 weeks.

Unfortunately, three months after the operation the patient presented herself again in the ED with acute pain in her left hip for 2 days and an inability to walk. There was no preceding trauma and there were no signs of an infection. The X-ray in the ED showed medial migration of the lag screw (Figure 5).

It was decided to remove the Gamma nail and replace it by a cemented total hip arthroplasty (Exeter Stem, Rimfit cup, Stryker) during the same session. Intraoperatively, the fracture appeared consolidated and there were no signs of infection.

**Figure 6:**
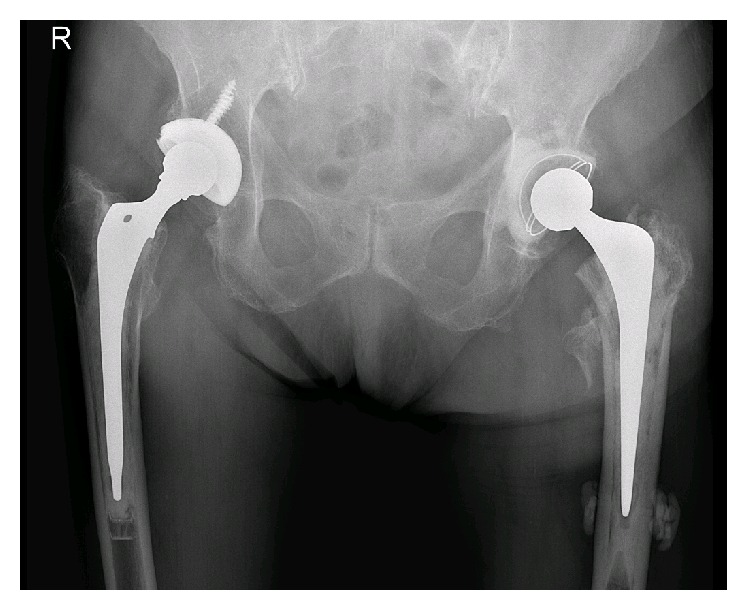
Anterior-posterior X-ray showing the cemented total hip arthroplasty after removal of the Gamma nail.

Patient was allowed to bear full weight one day after surgery. The X-ray showed a good position of the total hip arthroplasty (Figure 6). All the cultures that were taken during the removal of the Gamma nail showed no growth of bacteria.

Despite admission into the coronary care unit (CCU) for 1 day after the procedure for continuous monitoring of the cardiac rhythm, the rehabilitation was uneventful. After 15 days, the patient returned to the rehabilitation center for further recovery.

## 3. Discussion

Medial migration of the lag screw has by our understanding only been described in a few other case reports. And there has been only one other report in which the medial migration of the lag screw occurred later than in our case. We analyzed 6 case reports and several biomechanical and laboratory studies to offer an overview of the current literature on this topic and to determine the unexpected cause of the migration in our case.

Tauber and Resch presented a case of an 84-year-old woman who had received intramedullary fixation of a trochanteric fracture by Gamma nailing [5]. They report medial migration of the lag screw into the pelvis with a sigmoid perforation after 8 weeks. They removed the Gamma nail and hip replacement with a total cemented long stemmed hip prosthesis was performed. The authors discussed the so-called Z-effect as well as a caput-collum-diaphysis angle (CCD) which is too small (<125°). Eventually they conclude that none of these causes are applicable to their case. And therefore the actual cause remains uncertain.

This Z-effect was first described by Werner-Tutschku and Lajtai and referred to a characteristic sliding of the neck screws in opposite directions during the postoperative weight-bearing period in intramedullary fixation of per- and subtrochanter fractures with a Proximal Femoral Nail (PFN) [6]. As a possible explanation for this effect, varus stress during weight bearing in combination with an unstable calcar is given. This causes a collapse of the fracture and sliding of the inferior neck screw. This phenomenon was successfully reproduced in a laboratory setting by Strauss and Kummer [7]. They confirmed the explanation given by Werner-Tutschku and Lajtai and found that migration is likely to occur when the compressive strength of the femoral head is much greater than the femoral neck. This is the case in fractures with significant medial cortex comminution. There is also a “reverse Z-effect,” which was described by Boldin et al., in which the superior neck screw moves to lateral instead of medial [8].

Weil et al. analyzed 8 clinical cases of medial migration of the femoral neck element into the pelvis. They also created a biomechanical model simulating this complication in intramedullary nailing. They tested 5 different nail designs including the Gamma 3 nail. They found that certain fracture patterns lead to nail toggling within the femoral canal. These fracture patterns include an unstable medial calcar and a deficient lateral buttress. They also found that when toggling was prevented no migration did occur [9]. The authors compare the effect they found with the previously mentioned Z-effect.

Takasago et al. presented a case of a 63-year-old female who was treated for a trochanteric fracture of the left hip with a short Gamma 3 nail [10]. There were no unusual events and the early postoperative course was uneventful. After 6 weeks, the patient returned to the emergency department with a 1-week history of progressive pain in her left hip. Radiographs showed displacement of the fracture and intrapelvic migration of the lag screw through the femoral head and the medial wall of the acetabulum. There were no intra-abdominal injuries and a 2-stage revision of the Gamma nail into a total hip arthroplasty was performed. In their discussion, they state that the Z-effect is a more appropriate description for 2-screw devices, like the Proximal Femoral Nail (PFN). However, they acknowledge that a similar phenomenon can occur with a single femoral head fixation element. They hypothesize that in their case the femoral head could spin because the set screw had not been inserted appropriately to control the lag screw. As a result of this and insufficient reduction of the fracture, during repeated axial loading toggling of the nail within the femoral canal took place. This leads to medial migration of the lag screw, similar to what is seen in the Z-effect.

Li et al. presented a case of a 77-year-old female who suffered from an intertrochanteric hip fracture for which she was treated with a short Gamma 3 nail. Intraoperatively, there were no complications and the postoperative course was normal up until the 10th week of follow-up visit. Even though the patient was without complaints they report that the lag screw along with the short IM nail construct had migrated medially through the femoral head and through the medial wall of the acetabulum. Subsequently, the patient underwent revision surgery with removal of the lag screw and placement of a shorter lag screw. During this revision there was an additional cannulated screw placed which was located anterior to and parallel to the lag screw. The procedure was successful and the postoperative course was uneventful. They theorized that over time with compression of the fracture and dynamization of the nail in the setting of an unstable fracture pattern there was toggling of the nail within the intramedullary canal which led to the medial migration of the lag screw during repeated axial loading [11]. Thus, the same mechanism was explained by Weil et al.

Flint et al. present a case of intrapelvic migration of a Gamma nail in an 82-year-old woman, 7 months after fixation of an unstable pertrochanteric fracture. There were no intrapelvic structures compromised. The Gamma nail and lag screw were removed and replaced by an uncemented total hip arthroplasty. In their discussion they mention certain risk factors for femoral head and acetabular penetration of Gamma nail lag screws. These include damage to the femoral head by overreaming, malpositioned hardware, iatrogenic intraoperative medial displacement of lag screw, and avascular necrosis of the femoral head. Other mechanical reasons, include shearing and torsional muscle forces, failure to restore a stable medial cortical buttress, lateral buttress deficiency, unstable fracture patterns, or loss of posteromedial support. Furthermore, causes like lag screw interface dysfunction such as binding of the lag screw, axial loading in varus, inadequate lag screw length, nail toggling, and additional trauma after fixation have to be considered [12].

Heineman et al. reported an 83-year-old female who had been treated with a Gamma nail for an unstable intertrochanteric fracture of the right hip [13]. After three weeks she returned to the emergency department as a result of diminished walking ability. The pelvic X-ray showed an intra-abdominally migrated lag screw. Therefore, the Gamma nail was removed and revised to a total hip arthroplasty in two stages, because of fear of infection and abdominal trauma due to the migrated lag screw. The operation was without complication, and the postoperative course was complicated by a dislocation of the hip. In their discussion they mention that the migration could be the result of failure of the set screw or insufficient fastening of the set screw. A set screw has been designed to couple the lag screw to the nail, allowing controlled collapse without rotation of the screw within the nail. This, however, does not prevent rotation of the femoral head and neck around the screw. They also refer to the Z-effect but deem it more likely that in their case the set screw had not been fastened enough.

In the case presented by Thein et al., medial migration of the Gamma nail occurred after five weeks. The computed tomography (CT) scan showed that the lag screw migrated through the acetabulum and touched a branch of the left internal iliac artery. This branch was embolized and the implant was removed and replaced by a total hip arthroplasty. They mention different causes for the medial migration, many of which have already been discussed above. They also refer to the position of the tip of the lag screw as a possible predictor for medial migration [14]. The biomechanical analysis of Kuzyk et al. shows that the femoral lag screw is best positioned inferiorly on the anteroposterior radiograph and centrally on the lateral radiograph [15]. In the end they conclude that insufficient fastening of the set screw was most likely the cause for medial migration in their case.

Summarizing, different causes for this complication in the use of the Gamma nail are mentioned in the literature. Most frequently it is referred to a phenomenon similar to the Z-effect that occurs in two screw devices. This phenomenon seems to be a realistic cause and is reproduced in a laboratory setting. Another frequently mentioned cause is insufficient or inadequate tightening of the set screw. Other causes and recommendations comprise an adequate CCD angle and good placement of the tip of the lag screw in the femoral head.

Retrospectively, we hypothesize that in our case the set screw was not inserted correctly or that it might have loosened over time. Because there was sufficient reduction of the fracture with enough posteromedial support, there was little to none toggling of the Gamma nail possible. This might be the explanation for the late occurrence of the medial migration of the lag screw in our case.
